# Isoflurane Post-conditioning Ameliorates Cerebral Ischemia/Reperfusion Injury by Enhancing Angiogenesis Through Activating the Shh/Gli Signaling Pathway in Rats

**DOI:** 10.3389/fnins.2019.00321

**Published:** 2019-04-09

**Authors:** Li Peng, Jiangwen Yin, Mingyue Ge, Sheng Wang, Liping Xie, Yan Li, Jun-qiang Si, Ketao Ma

**Affiliations:** ^1^Department of Anesthesiology, First Affiliated Hospital, School of Medicine, Shihezi University, Shihezi, China; ^2^Division of Life Sciences and Medicine, Department of Anesthesiology, First Affiliated Hospital of USTC, University of Science and Technology of China, Hefei, China; ^3^Department of Physiology, School of Medicine, Shihezi University and The Key Laboratory of Xinjiang Endemic and Ethnic Diseases, Shihezi, China

**Keywords:** Sonic hedgehog, glioblastoma (Gli), isoflurane, post-conditioning, angiogenesis, cerebral ischemia/reperfusion

## Abstract

**Background:** Stroke is the second leading cause of death worldwide. Angiogenesis facilitates the formation of microvascular networks and promotes recovery after stroke. The Shh/Gli signaling pathway is implicated in angiogenesis and cerebral ischemia-reperfusion (I/R) injury. This study aimed at investigating the influence of isoflurane (ISO) post-conditioning on brain lesions and angiogenesis after I/R injury.

**Methods:** Adult male Sprague-Dawley rats were subjected to middle cerebral artery occlusion (MCAO), 1.5 h occlusion and 24 h reperfusion (MCAO/R). The ISO *post-conditioning* group (ISO group) received 1 h ISO post-conditioning when reperfusion was initiated. Neurobehavioral tests, TTC staining, HE staining, Nissl staining, TUNEL staining, immunofluorescence (IF), immunohistochemistry (IH) and Western blot were performed to assess the effect of ISO after I/R injury.

**Results:** ISO post-conditioning resulted in lower infarct volumes and neurologic deficit scores, higher rate of neurons survival, and less damaged and apoptotic cells after cerebral I/R injury in rats. Meanwhile, ISO post-conditioning significantly increased the expression levels of vascular endothelial growth factor (VEGF) and CD34 in the ischemic penumbra, relative to that in the Sham and I/R groups. However, cyclopamine, the specific inhibitor of the Sonic hedgehog (Shh) signaling pathway, decreased the expression levels of VEGF and CD34, and counteracted the protective effects of ISO post-conditioning against I/R injury in rats.

**Conclusions:** ISO post-conditioning enhances angiogenesis *in vivo* partly via the Shh/Gli signaling pathway. Thus, Shh/Gli may represent new therapeutic targets for aiding recovery from stroke.

## Introduction

Stroke is characterized by high morbidity, disability and mortality, with ischemic stroke being the most frequent prevalence (Feigin et al., 2014). Restoring cerebral perfusion in the ischemic area and saving the neurons in the ischemic penumbra as soon as possible are the central issues and goals of cerebral infarction treatment. However, the recovery of blood supplied to the ischemic area may also induce further tissue damage and dysfunction, which is known as ischemia-reperfusion (I/R) injury (Eltzschig and Eckle, 2011). Therefore, reduction cerebral I/R injury has become the key in the therapy of ischemic cerebrovascular disease.

Angiogenesis is the process of growing new capillaries from existing blood vessels by sprouting, proliferating, migrating and matrix remodeling of endothelial cells during tissue growth and development or pathophysiological conditions, such as trauma repair, tumor growth and tissue ischemia (Seevinck et al., 2010). Studies have shown that angiogenesis is a prominent feature in the ischemic penumbra after cerebral infarction, with consequent increasing microvessel density and improving oxygen and nutrient supply to the ischemic penumbra, thereby remodeling the post-ischemic microvascular networks, reducing the cerebral infarction volume and extending the survival time of stroke patients (Liu et al., 2014; Adamczak and Hoehn, 2015). Emerging evidence has also proven that angiogenesis protects brain from I/R injury and may be a treatment strategy to improve functional recovery after stroke (Doeppner et al., 2015; Jiang et al., 2016).

ISO is a common volatile anesthetic used extensively in the clinic and influences angiogenesis (Luo et al., 2015). Moreover, ISO is routinely used to anesthetize animals during animal model surgeries and induces neuroprotection in rats (Li and Zuo, 2011). In recent years, an increasing number of studies have shown that ISO protects against ischemic brain injury (Kim et al., 2015; Zhao et al., 2016; Cheon et al., 2017). We demonstrated previously that 1.5% ISO post-conditioning provides the best neuroprotection in rats after focal cerebral I/R injury (Wang et al., 2016). However, the brain protection mechanism of ISO post-treatment impacts angiogenesis has not yet been fully elucidated.

Sonic hedgehog induces robust angiogenesis (Dutzmann et al., 2017) and is activated to ameliorates cerebral ischemic injury by improving neurological function, promoting neurogenesis, and axonal remodeling (Zhang et al., 2013). In the canonical Hedgehog (Hh) signaling pathway, Shh links to Patched (Ptch) receptor in an autocrine or paracrine manner, relieving inhibition of Ptch on Smoothed (Smo), allowing Glis to enter the nucleus and initiating the expressions of target genes (Alvarez-Buylla and Ihrie, 2014). Moreover, a variety of experimental studies have shown that the Shh signaling pathway is critical to I/R injury recovery (Meng et al., 2016; Ge et al., 2017; Zeng et al., 2017).

Considering these findings, we hypothesized that the Shh signaling pathway is involved in ISO-induced angiogenesis after ischemic stroke. Thus, we investigated whether 1.5% ISO post-conditioning can activate the Shh signaling pathway, induce cerebral angiogenesis and ameliorate I/R injury in a rat model of MCAO/R.

## Materials and Methods

### Animals

All animal procedures in this study were approved by the Animal Experimental Committee of the First Affiliated Hospital of the Medical College, Shihezi University, and proceeded in accordance with the National Institutes of Health Guide for the Care and Use of Laboratory Animals. Forty adult male Sprague-Dawley rats (220–280 g) were supplied by the Experimental Animal Center of Shihezi University, China.

### Model Establishment and Animal Grouping

ISO was delivered with the vehicle air (30% O_2_ and 70% medical air) using an agent-specific vaporizer (Datex Ohmeda, USA) (Altay et al., 2012). Immediately at the beginning of reperfusion, rats were placed into the inhalation anesthesia device (a home-made patented product, patent number: ZL201520074763.0) for 1 h after the ISO concentration reached a steady level of 1.5% (Yuan et al., 2018). Concentrations of ISO was measured continuously using the anesthetic gas monitor (Drager Vamos, Germany).

Rats were randomly assigned into five groups: animals received sham operation and equal volume of DMSO (Sham group), MCAO rats treated with equal volume of DMSO group (I/R group), MCAO rats treated with equal volume of DMSO and 1.5% ISO post-conditioning (ISO group), MCAO rats treated with the Smo inhibitor cyclopamine (10 mg/kg) dissolved in DMSO intraperitoneally (Alvarez et al., 2011) after ISO post-conditioning group (ISO + CYC group), and MCAO rats treated with cyclopamine (10 mg/kg) dissolved in DMSO intraperitoneally group (I/R + CYC group). The DMSO and inhibitor were administered intraperitoneally at 30 min prior to ischemia. In the sham-operated group, the right common carotid artery, internal carotid artery, and external carotid artery were isolated, but no ligation was performed. The treatment to the ISO + CYC group was identical to the ISO group, except that the rats received an intraperitoneal injection of specific SMO inhibitor cyclopamine dissolved in DMSO. Rats in the Sham group, I/R group and I/R+CYC group were placed in the same chamber perfused only by vehicle air without isoflurane.

Rats were anesthetized with ketamine hydrochloride (60 mg/kg, intraperitoneally), and the procedure was performed as described by Li et al. (2014). A 3–0 monofilament nylon suture was introduced through the right common carotid artery into the internal carotid artery until resistance was encountered. It was advanced 18–20 mm, and a silk thread was tied to the right common carotid artery. The filament was withdrawn after 1.5 h of occlusion to allow reperfusion. Rats in the sham group were subjected to the same procedure without the filament advanced to the middle cerebral artery origin. Rats that died, experienced surgical failure, or underwent subarachnoid hemorrhage were excluded from this study.

### Evaluation of Neurologic Deficit Scores

To determine neurological function, we obtained the following modified Longa scores (Ding et al., 2002) at 24 h after MCAO: no deficits (value = 0); difficulty in fully extending the contralateral forelimb (value = 1); 2, unable to extend the contralateral forelimb (value = 2); mild circling to the contralateral side (value = 3); severe circling (value = 4); and falling to the contralateral side (value = 5). The treated rats were evaluated by an observer blinded to the experimental grouping.

### Measurement of Infarct Volumes

Rats were deeply anesthetized and sacrificed by guillotine after the evaluation of neurological deficit score test. Rats brains were removed and slices were taken at 2 mm intervals rapidly. The slices were stained using 2% 2,3,5-triphenyltetrazolium chloride (TTC) (Sigma, USA) for 30 min at 37°C. The stained slices were photographed after fixed in 4% paraformaldehyde (PFA) (Sigma, USA) for 24 h. The infarct volume was measured as previously described (Tatlisumak et al., 1998) and analyzed using the Image-Pro Plus 6.0 software (Media Cybernetics, USA).

### Hematoxylin-Eosin (HE) Staining

The rats were deep anesthetized and then transcardially perfused with normal saline followed by 4% PFA(Sigma, USA)at 24 h after MCAO. Brains were removed and fixed in 4% PFA for 24h before embedded in paraffin. Four-micrometer-thick sections were cut in the microtome (KEDEE, China), dewaxed in xylene, and then dehydrated in alcohol. Then, the sections were stained with hematoxylin for 3 min and eosin for 1 min. Finally, the sections were observed under a light microscope (Olympus, Japan) to assess brain injury in the penumbra of the ischemic cortex. The paraffin sections were also utilized for further experiments.

### Nissl Staining

Paraffin sections were deparaffinized in xylene and dehydrated in gradations of 70, 75, 90, 95, and 100% ethanol in water. The sections were stained with thionine (Solarbio, China) for 1 h at 37°C. Cells morphology of the cerebral cortex was observed under a microscope (Olympus, Japan) to assess brain damage (Gong et al., 2012). The number of surviving neurons was quantified by an observer without knowledge of the experiment.

### Terminal Deoxynucleotidyl Transferase dUTP Nick End Labeling (TUNEL) Staining

TUNEL assay was performed using the *In Situ* Cell Death Detection Kit (Roche, Germany) in accordance with the manufacturer's instruction after a standard histochemical procedure to determine the density of TUNEL-positive cells in the cerebral cortex. The apoptotic index (AI) was the number of apoptotic nuclei in 100 nuclei. AI = (number of TUNEL-positive cells/ total cells) × 100%.

### Immunohistochemistry (IH) Staining

Sections were immersed in citrate buffer (pH 6.0) and microwaved for 20 min after a standard histochemical procedure. Then, the sections were rinsed in phosphate-buffered saline (PBS) three times for 3 min before treated with 3% hydrogen peroxide for 15 min. After blocking with PBS containing 0.3% Triton X-100 and 10% bovine serum albumin, sections were incubated with anti-Shh (1:100, Santa Cruz Biotechnology, USA) or anti-CD34 (1:100, Santa Cruz Biotechnology, USA) for 60 min at 37°C. After washing with PBS three times for 3 min, the sections were reacted with the corresponding secondary antibodies for 30 min at 37°C. The sections were incubated with fresh diaminobenzidine (DAB) (Maixin, China) for 5 min and then with enough Hematoxylin for 1 min. The stained cells were observed under a microscope (Olympus, Japan) and then counted using the Image-Pro Plus 6.0 software (Media Cybernetics, USA).

### Immunofluorescence (IF) Staining

Paraffin sections for IF were stained with anti-Shh (1:100, Santa Cruz Biotechnology, USA), anti-Gli1 (1:100, Santa Cruz Biotechnology, USA), anti-VEGF (1: 100, Abcam, UK) and anti-CD34 (1: 100, Santa Cruz Biotechnology, USA) overnight at 4°C. Then the sections were washed with PBS and then incubated with the secondary antibody FITC-labeled goat anti-mouse antibody (1:50, ZSGB-BIO, China) at 37°C for 1 h. Next, the cellular nuclei were stained with propidium iodide (PI) solution for 5 min in the dark. Finally, the images were captured using a confocal laser scanning microscope (Olympus, Japan) and the mean fluorescence density was analyzed by the Image-Pro Plus 6.0 software (Media Cybernetics, USA).

### Western Blot Analysis

The proteins were isolated from the ischemic cortex tissue using lysis buffer (Beyotime, China), and protein concentrations were examined using a BCA protein assay kit (Beyotime, China). The proteins were separated by sodium dodecyl sulfate-polyacrylamide gel electrophoresis (SDS-PAGE) and transferred onto polyvinylidenedifluoride (PVDF) membranes. After blocking with 5% skimmed milk, the membranes were incubated with primary antibodies: anti-Shh, anti-Ptch, anti-Smo, anti-Gli1, anti-CD34 (1:1000, Santa Cruz Biotechnology, USA), and anti-VEGF (1:1000, Abcam, UK) overnight at 4 °C. After rinsing four times in TBST buffer for 5 min, the membranes were incubated with secondary antibodies (1:20000, ZSGB-BIO, China) at room temperature for 2 h and then treated with ECL reagent (ThermoFisher, USA) to detect protein expression levels. The protein bands were quantitatively analyzed using the Image J software (Rawak Software Inc., Germany).

### Statistical Analysis

All data are expressed as mean ± SD. Analysis among multiple groups was carried out by ANOVA. Student's *t*-test was used for two-groups comparison. Statistical analyses were conducted with SPSS 19.0 software and *P* < 0.05 was considered to be statistically significant.

## Results

### Cyclopamine Cancels the Protective Effects of Iso Post-conditioning on I/R Injury in Rats

**Figure 1:**
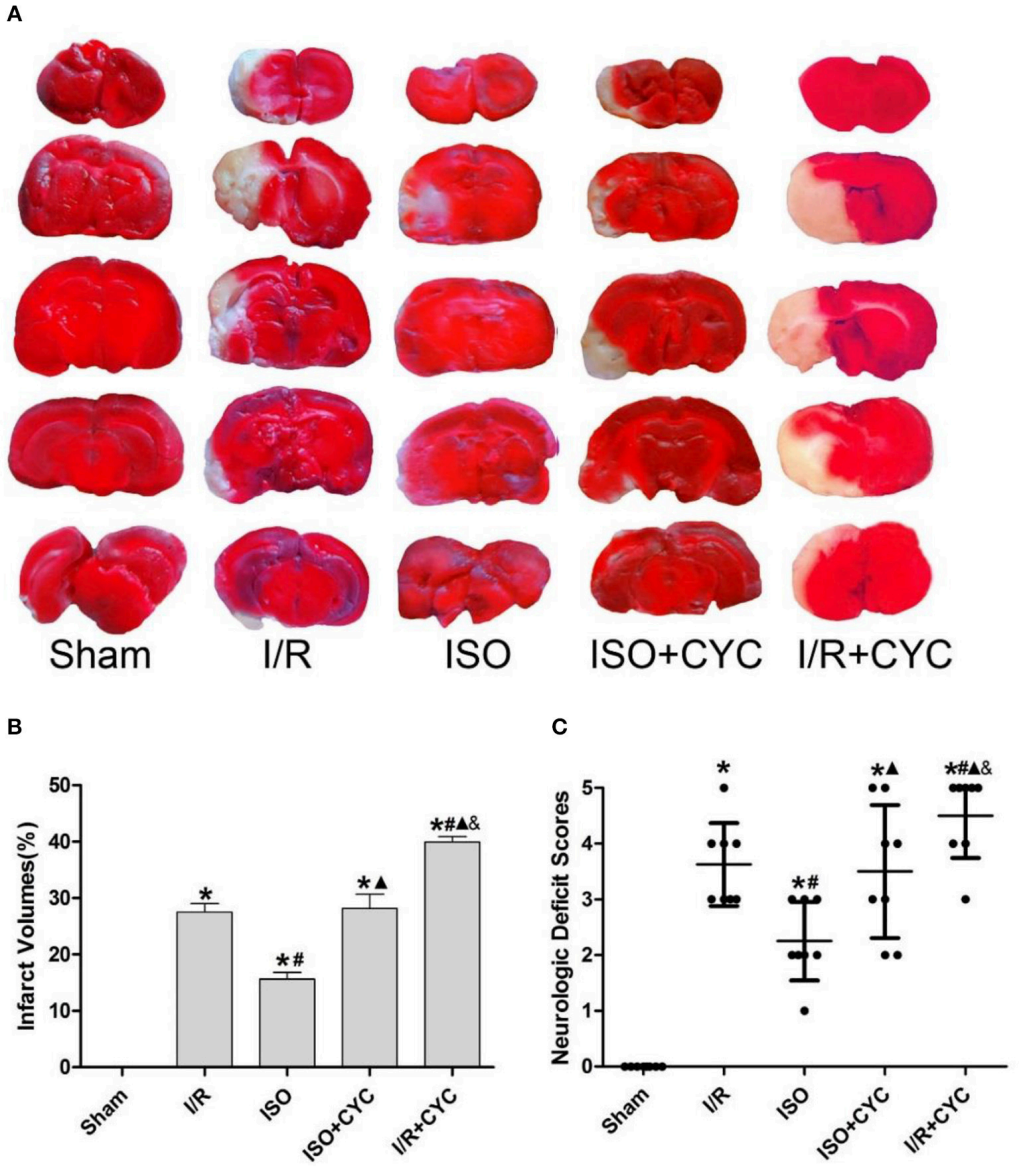
Cyclopamine inhibits the improved infarct volumes and neurologic deficit scores provided by ISO post-conditioning. **(A)** showed infarct volumes were assessed by TTC. Red represented normal tissue and white represented infarct tissues. **(B)** showed the quantitative data of infarct volumes. **(C)** showed neurological function scores with the modified Longa score. Data are presented as the mean ± SD (*n* = 8). ^*^P < 0.05 vs. Sham; #*P* < 0.05 vs. I/R; ▴*P* < 0.05 vs. ISO; &*P* < 0.05 vs. ISO + CYC.

No infarct volume and neurological deficit were observed in the sham group. Post-conditioning with ISO significantly reduced the infarct volumes and improved neurologic deficit scores compared with the I/R group at 24 h after MCAO/R injury in rats (15.66 ± 1.14, 2.25 ± 0.71 in the ISO group vs. 27.52 ± 1.50, 3.63 ± 0.74 in the I/R group, *P* < 0.05). However, the effects of ISO on infarct volumes and neurologic deficit scores were attenuated by the Smo inhibitor cyclopamine (15.66 ± 1.14, 2.25 ± 0.71 in the ISO group vs. 28.13 ± 2.58, 3.50± 1.20 in the ISO + CYC group, *P* < 0.05). Moreover, infarct volume and neurological deficit scores were the highest in the MCAO/R rats treated with cyclopamine (39.92 ± 0.97, 4.50 ± 0.76 in the I/R + CYC group; *P* < 0.05) (Figure 1).

**Figure 2:**
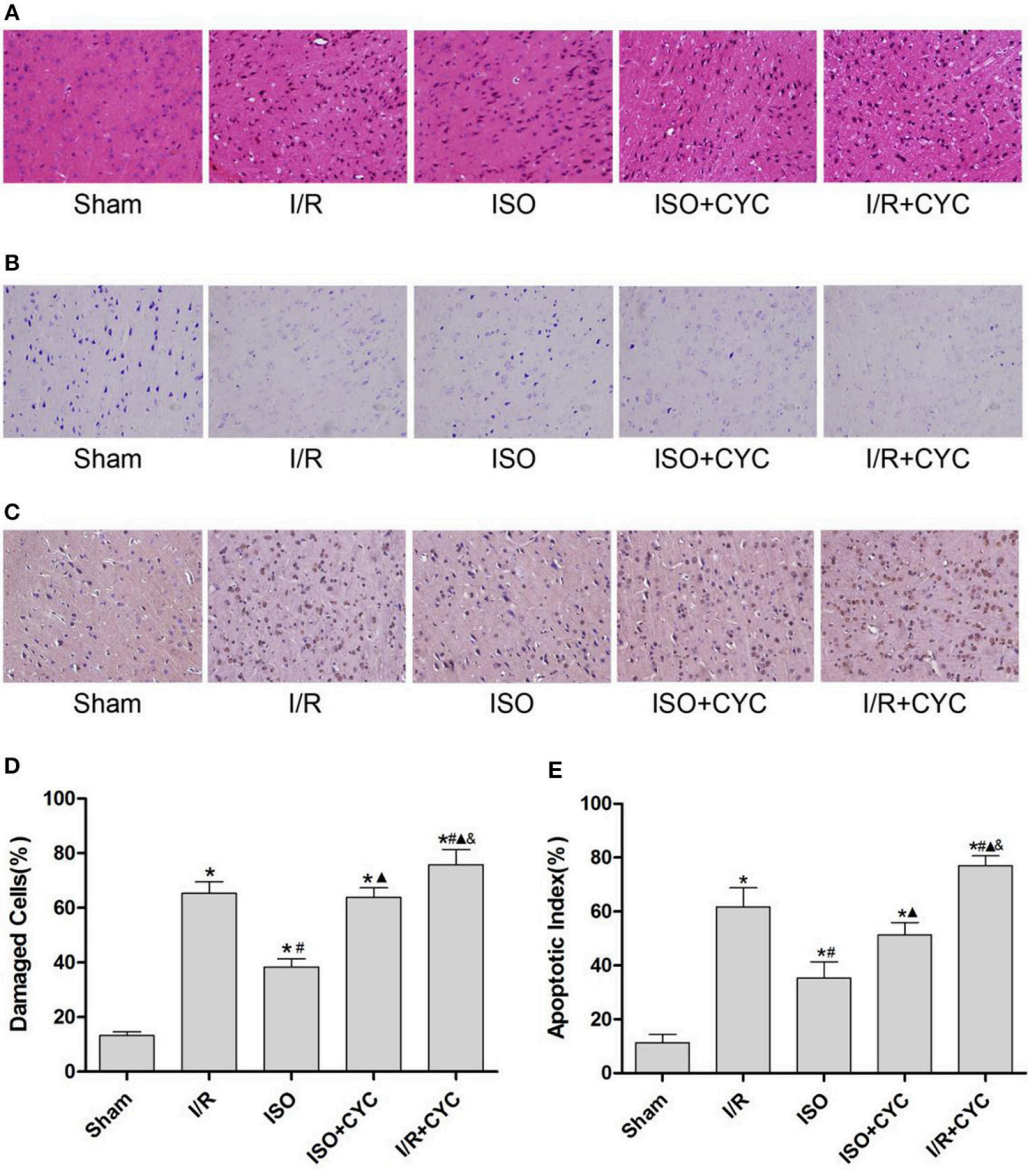
ISO post-conditioning has protective effects on I/R injury and cyclopamine cancels the effects of ISO in the ischemic penumbra. **(A)** showed the cells morphology by HE staining. **(B)** Nissl staining of the surviving cells. **(C)** showed the TUNEL-positive cells. **(D)** showed the percentage of damaged cells. **(E)** showed the apoptotic index. Data are presented as the mean ± SD (*n* = 3). Scale bars = 100 μm. *P < 0.05 vs. Sham; #*P* < 0.05 vs. I/R; ▴*P* < 0.05 vs. ISO; &*P* < 0.05 vs. ISO + CYC.

Next, we further investigated the effects of ISO post-conditioning and cyclopamine on I/R injury in the penumbra of the ischemic cortex by histopathological examination. The cells morphology in the I/R group displayed nuclear pyknosis, whereas those in the ISO group showed less damage after HE staining (38.30 ± 3.04 in the ISO group vs. 47.33 ± 4.26 in the I/R group, *P* < 0.05). However, the inhibitor cyclopamine attenuated the protective effect of ISO (38.30 ± 3.04 in the ISO group vs. 51.72 ± 4.24 in the ISO + CYC group, *P* < 0.05). Moreover, the percentage of damaged cells was the highest in the MCAO/R rats treated with cyclopamine (75.70 ± 5.68 in the I/R + CYC; P < 0.05) (Figures 2A,D).

The Sham group showed abundant cells with clear borders and Nissl bodies (104.00 ± 7.55). ISO post-conditioning significantly increased the surviving cells compared with the I/R group at 24 h after MCAO/R injury in rats (71.67 ± 2.52 in the ISO group vs. 47.33 ± 4.04 in the I/R group, *P* < 0.05). However, the effects of ISO on surviving cells were attenuated by cyclopamine (71.67 ± 2.52 in the ISO group vs. 59.33 ± 4.16 in the ISO+CYC group, *P* < 0.05). Moreover, the number of surviving cells in the penumbra of brain tissue was the lowest in the MCAO/R rats treated with the inhibitor (37.00 ± 3.61 in the I/R + CYC; *P* < 0.05) (Figure 2B, Table 1).

**Table 1:** Histological grades (HG) and neuronal density (ND) in the ischemic penumbra.

**Group**	**HG**				**ND**
	**0**	**I**	**II**	**III**	
Sham		6			104.00 ± 7.55
I/R			5	1	47.33 ± 4.04*
ISO			6		71.67 ± 2.52*#
ISO + CYC			4	2	59.33 ± 4.16*#▴
I/R + CYC			1	5	37.00 ± 3.61*#▴&

*The standard of HG: grade 0, no cell death; grade I, scattered cell death; grade II, mass cell death; grade III, almost complete cell death. ND was represented by the number of surviving pyramidal neurons per 1 mm^2^ length in the ischemic penumbra*.

The TUNEL-positive cells were the least in the sham group and significantly lower in the ISO group than in the I/R group (11.33 ± 3.06 in the Sham group, 35.33 ± 6.02 in the ISO group vs. 61.67 ± 7.09 in the I/R group, *P* < 0.05). However, cyclopamine attenuated the ISO's protective effect on the cells (35.33 ± 6.02 in the ISO group vs. 51.33 ± 4.51 in the ISO+CYC group, *P* < 0.05). Moreover, apoptotic index was the largest in the MCAO/R rats treated with cyclopamine (77.00 ± 3.61 in the I/R+CYC; *P* < 0.05) (Figures 2C,E).

These results indicated that ISO post-conditioning significantly ameliorated the brain I/R injury; the protective effects of ISO were remarkably inhibited with the administration of the inhibitor cyclopamine. Notably, the injury in the I/R rats treated with the inhibitor was more serious than in the other groups.

### ISO Post-conditioning Enhances the Activation of the Shh/Gli Signaling Pathway After Cerebral I/R Injury in Rats

**Figure 3:**
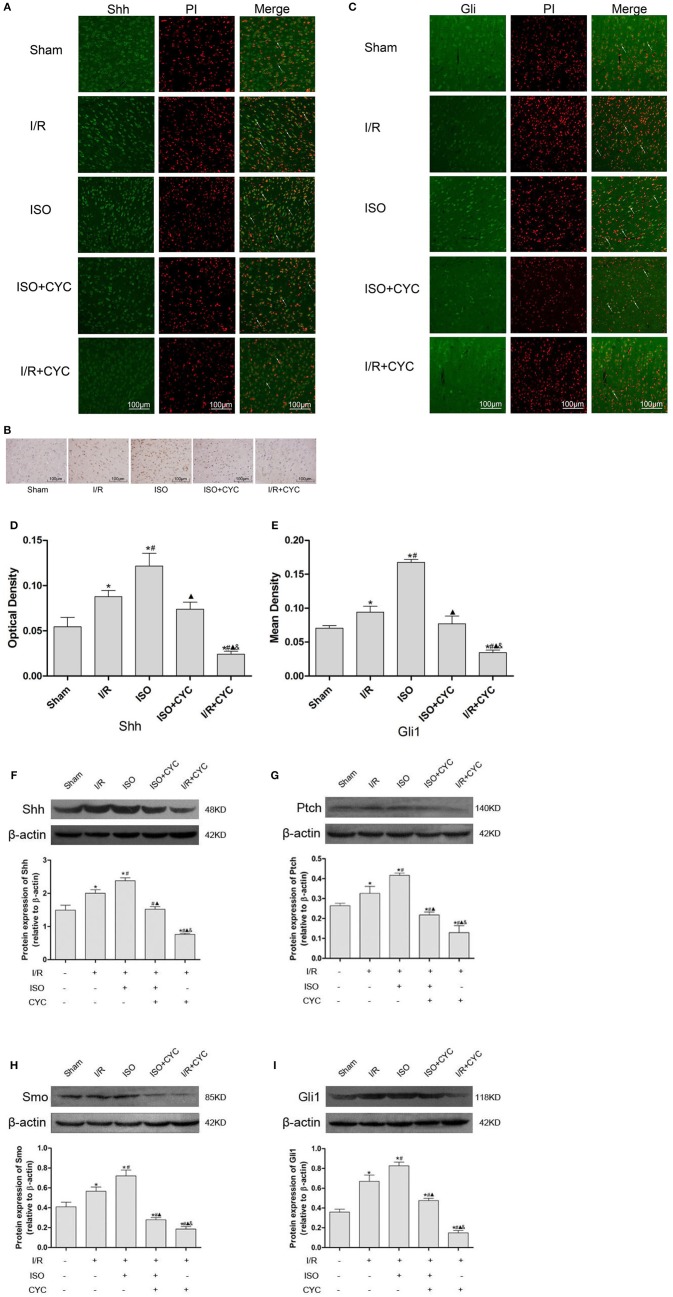
Expression of the Shh/Gli signaling pathway in the penumbra of the ischemic cortex in rats. **(A)** showed IF of Shh in the ischemic penumbra. **(B)** showed IH of Shh in the ischemic penumbra. **(C)** showed IF of Gli1 in the ischemic penumbra. **(D)** showed the optical density of Shh in each group. **(E)** showed the mean fluorescence density analysis of Gli1. **(F)** Proteins expression levels and Western blot analysis of Shh. **(G)** Protein expression levels and Western blot analysis of Ptch. **(H)** Protein expression levels and Western blot analysis of Smo. **(I)** Protein expression levels and Western blot analysis of Gli1. Data are presented as the mean ± SD (*n* = 3). Scale bars = 100 μm. *P < 0.05 vs. Sham; #*P* < 0.05 vs. I/R; ▴*P* < 0.05 vs. ISO; &*P* < 0.05 vs. ISO + CYC.

IF and IH staining showed that Shh was located in the cytoplasm (Figures 3A,B). Analysis of Shh optical density among all the groups showed that the expression level of Shh was low in the sham group (0.05 ± 0.01 in the Sham group). The expression level of Shh in the I/R group at 24 h after MCAO/R injury significantly increased, and ISO application obviously increased the expression compared with the I/R group (0.12 ± 0.01 in the ISO group vs. 0.09 ± 0.01 in the I/R group, *P* < 0.05). However, cyclopamine attenuated the expression of Shh (0.12 ± 0.01 in the ISO group vs. 0.07 ± 0.01 in the I/R + CYC group, *P* < 0.05). In addition, the expression level of Shh in the I/R + CYC group was the lowest among all the groups (0.02 ± 0.01 in the I/R + CYC group, *P* < 0.05) (Figure 3D).

Moreover, we analyzed the proteins expression levels of Shh, Ptch, Smo and Gli1 in the ischemic cortex by Western blot analysis. After I/R injury, the proteins levels of Shh in the I/R group markedly increased compared with those in the Sham group (1.49 ± 0.15 in the Sham group vs. 2.00 ± 0.11 in the I/R group, *P* < 0.05). Application of ISO further increased the expression compared with the I/R group (2.38 ± 0.09 in the ISO group vs. 2.00 ± 0.11 in the I/R group, *P* < 0.05). However, cyclopamine attenuated the expression levels (1.52 ± 0.08 in the ISO + CYC group, 0.76 ± 0.03 in the I/R + CYC group, *P* < 0.05) (Figure 3F).

In parallel, Western blot analysis also showed similar proteins expression trends of Ptch, Smo and Gli1 in the cortex. After I/R injury, the proteins were increased, and ISO further promoted their expression, whereas cyclopamine reduced the increased expression (Figures 3G–I).

### Shh/Gli Signaling Pathway May Mediate VEGF and CD34 Expression With ISO Post-conditioning After Cerebral I/R Injury

Finally, we determined whether the Shh/Gli signaling pathway is involved in the angiogenesis induced by ISO post-conditioning. IH, IF and Western blot were performed to examine the expression of VEGF and CD34.

**Figure 4:**
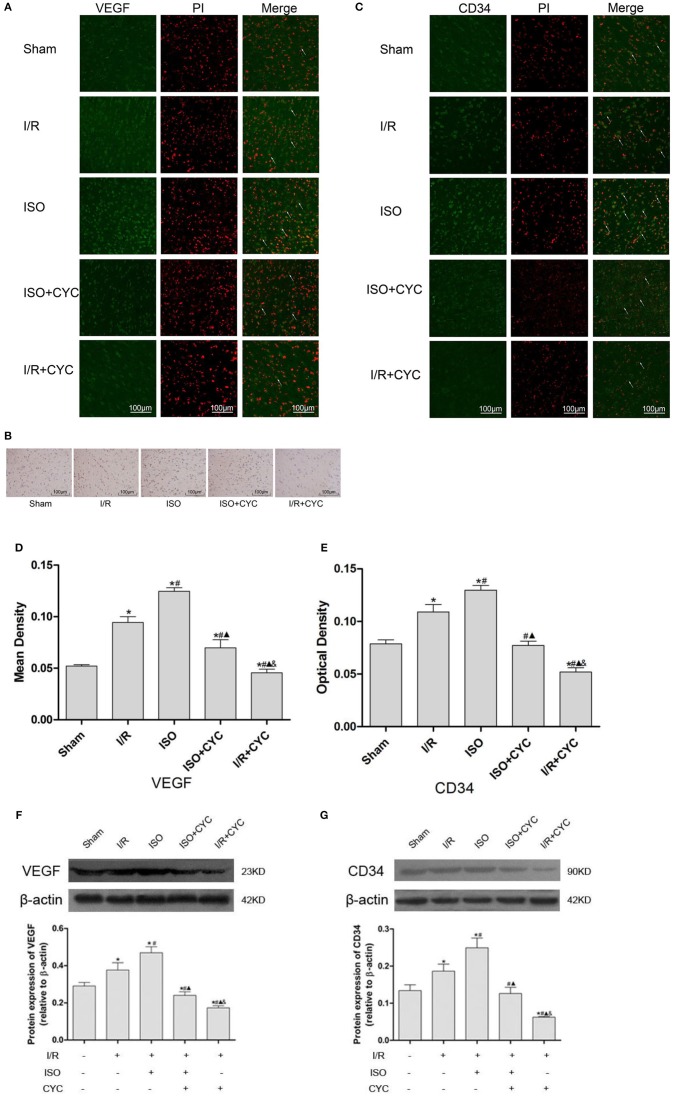
Expression of VEGF and CD34 in the penumbra of the ischemic cortex in rats. **(A)** showed IF of VEGF in the ischemic penumbra. **(B)** showed IH of CD34in the ischemic penumbra. **(C)** showed IF of CD34 in the ischemic penumbra. **(D)** showed the mean fluorescence density analysis of VEGF. **(E)** showed the optical density of CD34 in each group. **(F)** Protein expression levels and Western blot analysis of VEGF. **(G)** Proteins expression levels and Western blot analysis of CD34. Data are presented as the mean ± SD (*n* = 3). Scale bars = 100 μm. *P < 0.05 vs. Sham; #*P* < 0.05 vs. I/R; ▴*P* < 0.05 vs. ISO; &*P* < 0.05 vs. ISO + CYC.

IF staining showed that VEGF was located in the cytoplasm (Figure 4A). Analysis of VEGF's mean fluorescence density showed that the expression level of VEGF was low in the Sham group (0.05 ± 0.01 in the Sham group). The expression levels of VEGF in the I/R group at 24 h after MCAO/R injury significantly increased, and ISO application further increased the expressions compared with the I/R group (0.12 ± 0.01 in the ISO group vs. 0.09 ± 0.01 in the I/R group, *P* < 0.05) (Figure 4D). IH and IF staining showed that CD34 was localized in the cytoplasm (Figures 4B,C). After I/R injury, Gli1 significantly increased and ISO further promoted the increased expression (Figure 4E).

Moreover, Western blot analysis showed that the proteins expression levels of VEGF and CD34 had similar trends in the ischemic cortex. After I/R injury, the proteins levels of VEGF in the I/R group apparently increased compared with the Sham group (0.29 ± 0.02 in the Sham group vs. 0.38 ± 0.04 in the I/R group, *P* < 0.05). Application of ISO further increased the expression compared with the I/R group (0.47 ± 0.03 in the ISO group vs. 0.38 ± 0.02 in the I/R group, *P* < 0.05) (Figure 3F). Meanwhile, the proteins expression of CD34 also showed the same trend with VEGF (0.13 ± 0.02 in the Sham group, 0.17 ± 0.01 in the I/R group, 0.25 ± 0.03 in the ISO group, 0.13 ± 0.02 in the ISO + CYC group, 0.06 ± 0.01 in the I/R + CYC group, *P* < 0.05) (Figure 4G).

To further confirm the angiogenetic effect of the Shh/Gli signaling pathway activated by ISO, we administrated the Smo receptor inhibitor cyclopamine to the MCAO/R rats. Cyclopamine alone or in combination with ISO in the MCAO/R rats could depress the proangiogenic effect of ISO post-conditioning. Additionally, the expression levels of VEGF and CD34 were the lowest in the I/R + CYC group (Figure 4).

## Discussion

In this study, we demonstrated that ISO post-conditioning can considerably enhance angiogenesis via the Shh/Gli signaling pathway, thereby lowering infarct volumes and neurologic deficit scores, increasing surviving neurons, and minimizing damaged and apoptotic cells after cerebral I/R injury in rats. Moreover, our study indicated that ISO post-conditioning can modulate the production of VEGF and CD34, which may participate in the formation, remodeling, and maturation of microvascular networks in a Shh/Gli pathway-dependent manner.

Previous studies showed that the Shh/Gli signaling pathway governs a wide range of mechanisms in cell growth, survival, fate, and pattern almost every aspect of the vertebrate body plan (Varjosalo and Taipale, 2008). Recently studies have reported that Shh is involved in the repair of various tissue damages in adulthood and is up-regulated in ischemic models, such as skeletal muscle (Piccioni et al., 2014), myocardial tissue (Paulis et al., 2015), liver (Pratap et al., 2011), and brain tissue (Ding et al., 2013). In line with our results, exogenous administration of Shh can improve the behavioral scores and reduce the volume of cerebral infarction in rats with cerebral I/R injury; however, the specific inhibitor cyclopamine of the Shh pathway reverses the brain protection of Shh (Huang et al., 2013). In addition, the sonic hedgehog pathway agonist SAG (Jin et al., 2017) and purmorphamine (Chechneva et al., 2014) promote neuron regeneration and reduce apoptotic cell death in the ischemic cortex to restore neurological deficit.

Moreover, the activation of the Shh/Gli signaling pathway requires the participation of Ptch and Smo that regulates the expression levels of VEGF (Pola et al., 2001), which is critical for angiogenesis (Greenberg and Jin, 2013). The Hh signaling pathway mediates woven bone formation and angiogenesis in post-natal osteogenesis during stress fracture healing (Chechneva et al., 2014; Kazmers et al., 2015) The activated Shh/Gli signaling pathway promotes angiogenesis and protects against ischemic injuries in a wide variety of vascular cells, including endothelial, smooth muscle cells, and fibroblasts (Pola et al., 2001; Kusano et al., 2005; Palladino et al., 2011). Hh signaling, which is necessary for angiogenesis in ischemic muscle repair, can also be reactivated by Shh gene therapy (Renault et al., 2013). A recent study has shown that application of the Shh protein enhances VEGF expression, improves the microvascular density, and promotes angiogenesis, but these effects are blocked by cyclopamine *in vivo* and *in vitro* (Chen et al., 2017).

Therefore, we selected VEGF and CD34 for further research of the angiogenesis induced by ISO post-conditioning after cerebral I/R injury in rats. VEGF is a potent trigger of angiogenesis and shows blatant up-regulation hours after stroke (Jin et al., 2000). Studies showed that the infarct volume of the experimental group injected with exogenous VEGF after MCAO is reduced, and the brain water content is decreased, which indicates that VEGF exerts a direct protective effect on focal cerebral ischemia (Harrigan et al., 2003). CD34 is another endothelial antigen considered to be a reproducible, stable, and mature vascular endothelial marker that, can be used to assess microvascular density and an indirect marker of angiogenesis (Sidney et al., 2014).

A recent has study demonstrated that a 1.5 h isoflurane application nearly completely protects brain tissue from transient MCAO-induced injury and neurological deficits (Gaidhani et al., 2017). In our study, ISO post-conditioning for 1 h significantly increased the expression levels of VEGF and CD34, whereas the administration of cyclopamine dramatically suppressed the induction caused by ISO. Taken together, these observations indicate that ISO produces brain protection, however, cyclopamine blocks the Shh/Gli signaling pathway and attenuates the ISO-induced post-stroke angiogenesis.

Notably, in parallel to inducing angiogenesis, VEGF-increased vascular permeability leads to blood brain barrier injury and edema formation, which enhance the risk of hemorrhagic transformation (Greenberg and Jin, 2013). Consequently, appropriate timing and route of VEGF-A administration are important to obtain desirable results (Kaya et al., 2005). Some data reflect the non-canonical Shh pathways in the regulation of angiogenesis (Chinchilla et al., 2010). After oxygen-glucose deprivation, astrocyte stress produces Shh protein, whereas up-regulated Shh protein activates the RhoA/ROCK signaling pathway, which consequently affects angiogenesis *in vitro* (He et al., 2013).

In summary, this study is the first *in vivo* mechanistic study to demonstrated post-stroke ISO post-conditioning protects the brain from I/R injury by enhancing angiogenesis, which is associated with the Shh/Gli signaling pathway. Our findings lead to a better understanding of the beneficial effects of ISO against ischemic stroke and may provide a new avenue in stroke therapy.

## Ethics Statement

All animal procedures in this study were approved by the Animal Experimental Committee of the First Affiliated Hospital of the Medical College, Shihezi University, and proceeded in accordance with the National Institutes of Health Guide for the Care and Use of Laboratory Animals.

## Author Contributions

SW, LP, and JY conceived and designed the experiments; LP and MG conducted the experiment. LX, YL, JS, and KM provided assistance in experiment performing; LP and JY analyzed the data. LP and JY wrote the manuscript. All authors discussed and commented on the manuscript.

### Conflict of Interest Statement

The authors declare that the research was conducted in the absence of any commercial or financial relationships that could be construed as a potential conflict of interest.
